# Stoichiometric imbalances between terrestrial decomposer communities and their resources: mechanisms and implications of microbial adaptations to their resources

**DOI:** 10.3389/fmicb.2014.00022

**Published:** 2014-02-03

**Authors:** Maria Mooshammer, Wolfgang Wanek, Sophie Zechmeister-Boltenstern, Andreas Richter

**Affiliations:** ^1^Terrestrial Ecosystem Research, Department of Microbiology and Ecosystem Science, University of ViennaVienna, Austria; ^2^Institute of Soil Research, Department of Forest and Soil Sciences, University of Natural Resources and Life Sciences ViennaVienna, Austria

**Keywords:** Ecological stoichiometry, homeostasis, carbon/nutrient use efficiency, elemental imbalance, soil microbial communities, extracellular enzymes, mineralization, organic matter decomposition

## Abstract

Terrestrial microbial decomposer communities thrive on a wide range of organic matter types that rarely ever meet their elemental demands. In this review we synthesize the current state-of-the-art of microbial adaptations to resource stoichiometry, in order to gain a deeper understanding of the interactions between heterotrophic microbial communities and their chemical environment. The stoichiometric imbalance between microbial communities and their organic substrates generally decreases from wood to leaf litter and further to topsoil and subsoil organic matter. Microbial communities can respond to these imbalances in four ways: first, they adapt their biomass composition toward their resource in a non-homeostatic behavior. Such changes are, however, only moderate, and occur mainly because of changes in microbial community structure and less so due to cellular storage of elements in excess. Second, microbial communities can mobilize resources that meet their elemental demand by producing specific extracellular enzymes, which, in turn, is restricted by the C and N requirement for enzyme production itself. Third, microbes can regulate their element use efficiencies (ratio of element invested in growth over total element uptake), such that they release elements in excess depending on their demand (e.g., respiration and N mineralization). Fourth, diazotrophic bacteria and saprotrophic fungi may trigger the input of external N and P to decomposer communities. Theoretical considerations show that adjustments in element use efficiencies may be the most important mechanism by which microbes regulate their biomass stoichiometry. This review summarizes different views on how microbes cope with imbalanced supply of C, N and P, thereby providing a framework for integrating and linking microbial adaptation to resource imbalances to ecosystem scale fluxes across scales and ecosystems.

## INTRODUCTION

Soil microbial communities are key players in global biogeochemical cycles, regulating core ecosystem processes such as organic matter decomposition, soil C sequestration and nutrient recycling. Microbial decomposers release extracellular enzymes (EEs), which deconstruct plant macromolecules and ultimately liberate soluble substrates for microbial uptake (Schimel and Bennett, 2004). In turn, microbes use these substrates to fuel biomass production and EE synthesis (Moorhead et al., 2012). The amount of inorganic nutrients released into the ecosystem by mineralization depends on the relative C to the nutrient demand of the microorganisms, as well as the nutrient content of organic matter.

Microorganisms can be linked to these ecosystem processes through the theory of ecological stoichiometry, which has emerged as a powerful tool for studying the functioning of both aquatic and terrestrial ecosystems (Sterner and Elser, 2002). Soil is spatially and temporally heterogeneous, comprising chemically diverse compounds as well as harboring a vast diversity of microbes. Ecological stoichiometry uses elemental ratios and is thus a simplification of natural complexity, which can explain ecological dynamics simply by acknowledging chemical constraints on the metabolic and physiologic functions of organisms. Stoichiometric invariance (homeostasis) of organisms is a central concept in ecological stoichiometry to predict nutrient retention and recycling, as well as biomass production, from subcellular to ecosystem scales (Sterner and Elser, 2002). Stoichiometric homeostasis is defined as the degree to which organisms maintain a constant chemical composition despite variations in the chemical composition and availability of their resources (Sterner and Elser, 2002). Strictly homeostatic organisms have invariable C:N:P ratios, where changes in resource stoichiometry have no influence on their stoichiometry, whereas non-homeostatic organisms vary their elemental composition in response to changes in resource composition.

On a global scale, the stoichiometry of soil microbial biomass has been shown to be more constrained in range and variance compared to its resource, which implies that microbes are largely homeostatic in terms of their biomass C:N:P (Cleveland and Liptzin, 2007). Homeostatic regulation of microbial biomass composition constitutes the basis for the consumer-driven nutrient recycling theory (CNR; Sterner, 1990; Elser and Urabe, 1999; Sterner and Elser, 2002), according to which the elemental ratios of consumers and their resources determine the ratio of C:nutrient released through differential recycling of C and nutrients (N or P). This is of special interest in terrestrial ecosystems because microbial decomposers recycle C and N mainly as carbon dioxide and ammonium, respectively, contributing to soil respiration and soil N mineralization. Thus, the constraints on microbial growth and activity by the stoichiometric imbalance between microbial communities and their resource play a pivotal role in shaping ecosystem processes (Manzoni et al., 2010; Mooshammer et al., 2012), with the regulation of microbial homeostasis being an underlying, determining factor.

**FIGURE 1:**
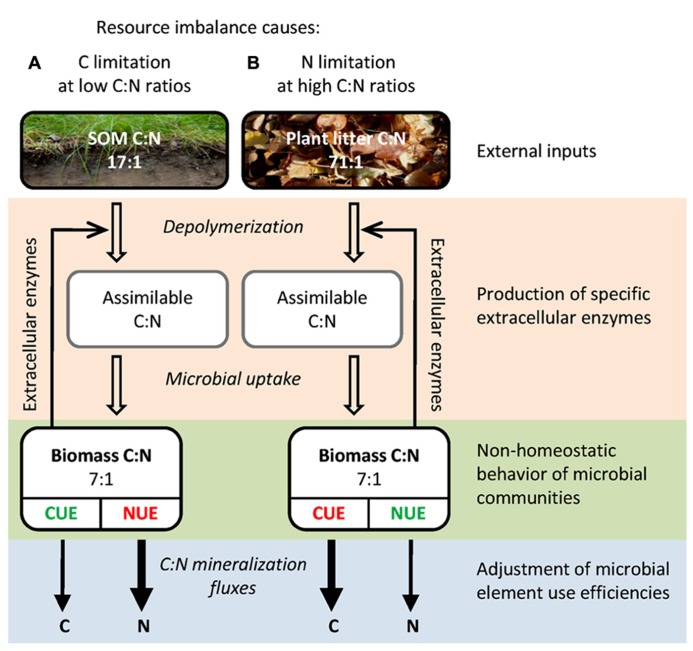
**Simplified schematic representation of important C:N components and fluxes during organic matter breakdown by litter or soil microbial communities.** The differences in C:N:P stoichiometry between **(A)** soil organic matter and **(B)** plant litter result in distinct elemental limitations for the respective microbial communities, with different implications for element mineralization fluxes. Mechanisms for microbial adaptation to these resource imbalances are indicated: input of external nutrients by N-fixing prokaryotes or by saprotrophic fungi, adjustment in the production of extracellular enzymes as C and nutrient acquisition strategy, non-homeostatic behavior of microbial communities and adjustment of microbial element use efficiencies. Given are global average molar C:N ratios (Yuan and Chen, 2009; Xu et al., 2013). The scheme is also applicable for P, by substitution of C:N ratios by C:P (plant litter, 3,055:1; SOM, 287:1; microbial biomass, 42:1; **Table 1**) as well as NUE by phosphorus use efficiency (PUE). Abbreviations: SOM, soil organic matter; CUE, carbon use efficiency; NUE, nitrogen use efficiency.

The key questions regarding stoichiometric imbalances, i.e., how soil microbes regulate their C:N:P homeostasis and how this in turn affects the processing of organic matter, are still insufficiently understood. The aim of this review is to synthesize the current knowledge on the mechanisms that allow terrestrial microbial communities to thrive in a stoichiometrically imbalanced world. First, we review the spatial and temporal variability of resource stoichiometry and its effect on microbial biomass stoichiometry. Second, we present a mechanistic framework of how soil microbial communities cope with resource imbalances (**Figure 1**), including (i) plasticity of microbial biomass C:N:P, (ii) production of EEs as C and nutrient acquisition strategy, (iii) adjustments in microbial element use efficiencies, and (iv) input of external nutrients by N-fixing prokaryotes or by saprotrophic fungi.

## STOICHIOMETRIC IMBALANCES BETWEEN RESOURCES AND SOIL MICROBIAL COMMUNITIES

For soil microbes the most important resources are plant necromass (wood, plant, and root litter), exudates and soil organic matter (SOM). Decomposable plant products (detritus, necromass, root exudates) exhibit much wider and more variable C:N:P ratios than microbes [see data compiled in **Table 1** and (Sistla and Schimel, 2012); all C:N:P ratios in this publication refer to molar ratios]. For instance, leaf litter C:N:P is globally constrained with a molar ratio of 3,007:45:1 (McGroddy et al., 2004), or 3,055:43:1, based on a larger dataset (Yuan and Chen, 2009). Leaf litter C:N:P, however, varies between ecosystems and biomes (McGroddy et al., 2004), and even more between plant species, life forms (e.g., grasses, forbs, shrubs, deciduous or evergreen trees) and broader phylogenetic groups (i.e., angiosperms, gymnosperms; Yuan and Chen, 2009). Therefore, in diverse multispecies plant communities, one can expect a large variability of C:N:P of leaf litter inputs. Root litter also has wide C:N:P ratios, with 4,184:43:1, although it remains unclear if systematic differences between species, ecosystems and biomes exist due to limited available data (Yuan et al., 2011). Nevertheless, molar C:N:P ratios of living fine root biomass clearly differ between biomes and decline exponentially with latitude, similar to those of leaf litter (Yuan et al., 2011). Woody debris has even wider C:N:P ratios than root litter due to very low N and P concentrations, with C:N:P ratios of boles and other coarse woody debris of 14,103:40:1 (**Table 1**; Harmon et al., 1986; Martinelli et al., 2000; Weedon et al., 2009). Wood of gymnosperms typically has wider C:N:P ratios (15,980:28:1) than that of angiosperms (6,122:28:1; Weedon et al., 2009), and tropical wood has a higher N:P ratio (93:1) than wood from temperate and boreal plants (27:1).

**Table 1:** Globally averaged element ratios in potential resources and in soil microbial biomass, and stoichiometric imbalances between resources and microbes calculated as the ratio of C:N_**resource**_ (or C:P, N:P) over C:N_**microbes**_ (or C:P, N:P).

Organic material	Molar C:N:P	C:N imbalance	C:P imbalance	N:P imbalance	Reference
Wood	14,103 (±2,898):40 (±13):1	50	336	7	Harmon et al. (1986), Martinelli et al. (2000), Weedon et al. (2009)
Dead roots	4,184 (±991):43 (±4):1	14	100	7	Yuan et al. (2011)
Leaf litter	3,055 (±181):43 (±1):1	10	73	7	Yuan and Chen (2009)
Soil organic matter	287 (±25):17 (±1):1	2	7	3	Xu et al. (2013)
Soil microbes	42 (±4):6 (±0.4):1				Xu et al. (2013)

Adding to this complexity, the availability and type of plant necromass varies seasonally (e.g., Bardgett et al., 2005). For instance, in temperate forests leaf litter fall occurs as a short pulse of a few weeks in autumn. In temperate ecosystems root turnover increases in late autumn and consequently root necromass becomes available for microbial decomposition in topsoil and subsoil (Drake et al., 2011). Root exudation also affects the availability of organic C relative to N and P. Root exudation peaks during the plant growing season (Phillips et al., 2011), changing the stoichiometry of available resources in the rhizosphere. Root exudates are thought to consist mainly of sugars and organic acids, with a minor contribution of amino acids (Marschner, 1995). Consequently, the C:N ratio of root exudates has been assumed to range between 50 and 100 (Drake et al., 2013). Although little empirical evidence exists, it is safe to suggest that C:N ratios of root exudates are much wider than those of SOM and microbial biomass. No data are currently available for root exudate P fluxes, but P concentration in exudates is presumably low. Our current knowledge of root exudate composition is notably limited and empirical data on exudate C:N:P fluxes are urgently needed to better define its effect on decomposition processes in soil.

Soil organic matter, which is the result of microbial decomposition of plant necromass, is another major resource available for heterotrophic microorganisms. Microbial residues play an important role in the formation of SOM, as plant resources are converted to microbial biomass during decomposition, comprising a large proportion of SOM (e.g., Simpson et al., 2007; Miltner et al., 2009). Consequently, resource C:N:P ratios decline and the stoichiometric variability decreases with SOM processing. For instance, the C:N ratios of deeper soil horizons, which have been processed for longer, converge toward the C:N ratios of microbial biomass. Therefore, soils have narrower molar C:N:P ratios than plant litter, with a global soil average of 287:17:1 (Xu et al., 2013). Similar to other resources, SOM shows significant differences in C:N:P between ecosystem types and biomes (Xu et al., 2013). A meta-analysis of ~2400 soil profiles collected in China further showed that soil C:N, C:P, and N:P ratios decrease with soil depth (Tian et al., 2010a). Soil C:N ratios are significantly higher in organic soils than in mineral soils, increasing linearly with soil organic carbon (SOC) content (**Table 2**, dataset of Xu et al., 2013). Based on this relationship, a C:N ratio of 13.6 is predicted for soils with a SOC content of 1%, whereas a C:N ratio of 39.2 is predicted for high SOC soil (i.e., 50% SOC). Along the same line, soil C:P ratios are predicted to be 156 and 1,610 for low and high SOC soils, and soil N:P ratios to be 10.3 and 55.4, respectively (**Table 2**). Significant differences in soil C:N:P along the SOM decomposition continuum therefore become evident, despite soils being stoichiometrically more constrained than plant detritus. As a result of the spatio-temporal variability in the availability and type of plant detritus and SOM, terrestrial microbial communities must cope with large spatio-temporal variability in resource C:N:P ratios.

**Table 2:** Relationships between soil organic carbon (SOC) content, soil C:N:P and microbial biomass C:N:P.

Relationship	Equation	*n*	*R*	*R*^l2^	*P*	Estimate	
						1% SOC	50% SOC
Soil C:N vs. SOC (%)	Soil C:N = 13.1 + 0.523 × SOC	2135	0.577	0.333	<0.001	13.6	39.2
Soil C:P vs. SOC (%)	Soil C:P = 126 + 29.7 × SOC	528	0.650	0.423	<0.001	156	1611
Soil N:P vs. SOC (%)	Soil N:P = 9.42 + 0.920 × SOC	506	0.505	0.255	<0.001	10.3	55.4
Mic C:N vs. SOC (%)	Mic C:N = 8.13 + 0.041 × SOC	1108	0.076	0.005	0.012	8.2	10.2
Mic C:P vs. SOC (%)	Mic C:P = 64.2 + 0.730 × SOC	561	0.088	0.008	0.037	64.9	100.7
Mic N:P vs. SOC (%)	Mic N:P = 7.16 + 0.067 × SOC	440	0.084	0.007	0.079	7.2	10.5
Mic C:N vs. soil C:N	Mic C:N = 7.81 + 0.031 × soil C:N	1023	0.063	0.004	0.044	7.8	9.4
Mic C:N vs. soil C:N (log–log)			0.121	0.014	<0.001		
Mic C:P vs. soil C:P	Mic C:P = 66.5 + 0.015 × soil C:P	405	0.078	0.006	0.118	66.5	67.3
Mic C:P vs. soil C:P (log–log)			0.000	0.000	0.992		
Mic N:P vs. soil N:P	Mic N:P = 6.81 + 0.046 × soil N:P	294	0.102	0.010	0.081	6.9	9.1
Mic N:P vs. soil N:P (log–log)			-0.097	0.009	0.091		

*The respective equations are used to estimate element ratios for low-C soils (1% soil organic carbon, SOC) and high-C soils (50% SOC). Data from Xu et al. (2013).*

The range of biomass C, N, and P concentrations in soil microbial communities spans several orders of magnitude, although the linear and isometric relationships between these elements suggest a fixed, or at least a highly constrained, microbial C:N:P ratio (Cleveland and Liptzin, 2007; Hartman and Richardson, 2013). A meta-analysis of microbial biomass C:N:P showed remarkably constant molar ratios around 60:7:1 (Cleveland and Liptzin, 2007), and more recently, an area-weighted global soil microbial biomass C:N:P of 42:6:1 was reported based on a fourfold larger dataset (Xu et al., 2013). Although similarities in microbial elemental ratios among sites across large scales have been stressed by several authors, there is also evidence for some stoichiometric flexibility of microbial communities (Li et al., 2012; Fanin et al., 2013; Hartman and Richardson, 2013). Significant differences in microbial biomass elemental ratios were found between different ecosystems, e.g., forests and grasslands (Cleveland and Liptzin, 2007), and also between major biomes, as shown by wide microbial biomass C:N:P ratios in natural wetlands and tundra, in contrast to the narrow C:N:P ratios in boreal forests, croplands, pastures and deserts (Xu et al., 2013). Microbial C:N ranged between 4.5 and 12.5 (95% confidence boundaries for major biomes), microbial C:P between 24 and 275, and microbial N:P between 3.5 and 10.6 (excluding wetlands, Xu et al., 2013). In another meta-analysis, Hartman and Richardson (2013) also reported significant variations in microbial N:P ratios among sites with different vegetation and land use types, across soils and litter layers, and suggested that these differences might be linked to variation in size-dependent scaling relationships of biomass C:N and C:P (i.e., slight increases in the proportions of N and P with increasing soil microbial biomass C pools).

Biome- and ecosystem-level differences in microbial C:N:P ratios may be explained by co-variation with resource stoichiometry, or by soil type, soil pH, soil C content and/or latitude (mean annual temperature). Xu et al. (2013) found significant negative correlations between latitude and microbial C:N, N:P, and C:P, although low correlation coefficients indicate a weak effect of mean annual temperature on microbial stoichiometry. Soil pH was not found to affect microbial C:N:P ratios (Cleveland and Liptzin, 2007; Hartman and Richardson, 2013; Xu et al., 2013). Interestingly a re-analysis of the existing global dataset of Xu et al. (2013), presented in **Table 2**, shows that microbial biomass C:N and C:P, but not N:P, are positively correlated with SOC content. This points to indirect control of SOC content on microbial stoichiometry, i.e., soil C:N and C:P ratios increase with SOC content, and microbial C:N and C:P as well. Moreover, this pattern indicates that in soil with high SOC content and high C:N (C:P), N or P may become limiting while C availability is high, thereby increasing microbial biomass C:N:P. On a global scale, microbial C:N:P ratios were not (Cleveland and Liptzin, 2007; Hartman and Richardson, 2013), or only weakly, positively related to the respective soil element ratios (Xu et al., 2013; **Table 2**), indicating a large degree of stoichiometric homeostasis of soil microbial communities. Xu et al. (2013) reported a positive relationship between resource C:N and microbial biomass C:N (**Table 2**), though the relationship between resource C:N and microbial C:N is strongly dampened in relation to the variability in resource elemental ratios. For example, a resource C:N ratio of 10 would predict a microbial biomass C:N of 8.1, but a 10-fold higher resource C:N of 100 would only lead to a 1.3-fold increase in microbial biomass C:N to 10.9.

As a consequence of the relatively low variation in microbial biomass C:N:P, large C:N:P imbalances arise between different types of decomposable organic matter and decomposer communities (**Table 1**). We estimated the average global C:N imbalance – calculated as resource C:N divided by microbial biomass C:N – ranging from 2 (SOM) to values between 10 and 14 (leaf litter, dead roots), and up to 50 (wood). The C:P imbalances are higher than the C:N imbalances, ranging from 7 (SOM), to 73 (litter), 100 (root litter) and 336 (wood). Smallest imbalances are found for N:P, ranging from 3 in SOM to 7 in plant detritus (leaf litter, dead roots, and wood). These patterns in resource stoichiometry raise the question of how soil microbial communities cope with such large elemental imbalances.

## ADAPTATIVE MECHANISMS OF SOIL MICROBIAL COMMUNITIES TO STOICHIOMETRIC IMBALANCES

There are four main mechanisms for microbial decomposers to cope with elemental imbalances and thrive on substrates that do not meet their elemental demand for growth (**Figure 1**): First, microbes can adjust their biomass C:N:P ratios to meet the elemental composition of their substrates; second, they change the elemental composition of their immediate substrates by producing EEs that preferentially deconstruct polymers that meet their demand for C and nutrients; third, after substrate uptake, they mineralize and excrete the elements in excess of their demand by regulating their element use efficiencies; and fourth, diazotrophic bacteria and saprotrophic fungi may trigger the input of external N and P to decomposer communities. Below, we review these four mechanisms in more detail:

(1) *Non-homeostatic behavior of microbes reduces the stoichiometric imbalance to their resource.* As demonstrated above, small adjustments of microbial biomass C:N:P ratios to resource C:N:P do indeed occur, and may help as an adaptive mechanism of microbial communities to stoichiometric imbalances. Adjustment of microbial biomass C:N:P to resource C:N:P ratios can occur due to two main mechanisms: first, microbial storage of elements in excess, leading to a convergence between the biomass and resource stoichiometries, and second, shifts in microbial community structure and concomitant shifts in biomass stoichiometry. Whereas the first mechanism would be a physiological adjustment of the stoichiometry of microorganisms, requiring that microbes are non-homeostatic, the latter is not a true adjustment, as a community change may have very different reasons.

Despite the lack of information regarding changes in microbial C, N, or P storage, analyses of cultured organisms have shown that P storage in polyphosphates by bacteria and fungi is related to increased P availability (Kornberg, 1995; Achbergerova and Nahalka, 2011), and C storage, for example, in lipids (triacylglycerols, TAG, and poly-β-hydroxyalkanoates, PHA) and glucans (such as glycogen), can increase with C availability (Wilkinson, 1963; Lee, 1996; Wilson et al., 2010). However, glucan and PHA storage usually does not exceed a few percent (up to a maximum of 20%) of biomass. Unlike C and P, N has no specific storage pool beside small, intermittent accumulation of essential amino acids, and no significant N storage in microbes has been reported to date (e.g., Banham and Whatley, 1991). It rather seems that N is either directly incorporated into the biomass, or mineralized and excreted. Therefore, C:N ratios are not expected to change markedly due to storage of excess C or N. Under P-limited conditions, microbes are thought to be relatively homeostatic in regard to C:P and N:P ratios (Makino et al., 2003). However, it has recently been shown in decomposition experiments with tropical leaf litter (at low P availability) that microbial N:P ratios followed that of the soluble litter fraction (Fanin et al., 2013). When P is available in excess, microbes can become strongly non-homeostatic (Scott et al., 2012). In such case, luxury P consumption and cellular P storage in the form of polyphosphates has been reported (Kornberg, 1995). For example, accumulated polyphosphate can comprise up to 10–20% of yeast cells’ dry mass, thereby strongly affecting their C:N:P stoichiometry. However, whether P supply in excess of microbial demand really exists in natural terrestrial ecosystems remains unclear.

On the other hand, the stoichiometric plasticity of microbial communities may be generated by shifts in the dominance of strains of distinct stoichiometry. Bacteria in general exhibit lower C:N ratios than fungi (Strickland and Rousk, 2010), and fast-growing microbes (copiotrophs, r-strategists) have been suggested to exhibit lower biomass C:N:P ratios (higher nutrient requirements) than slow-growing ones (oligotrophs, K-strategists; Elser et al., 2003; Fierer et al., 2007). Therefore, changes in fungal: bacterial ratios and shifts in the dominance of r- or K-strategists are expected to result in concomitant shifts in microbial biomass C:N:P ratios. Moreover, fast- and slow-growing microorganisms do not only exhibit distinct biomass stoichiometries, but also the requirement for stoichiometric homeostasis might vary with their growth rates, with tight requirements for fast-growing and relaxed requirements for slow-growing microorganisms (Egli, 1995; Fierer et al., 2007). Recently, Fanin et al. (2013) showed that a non-homeostatic behavior of microbial biomass was due to shifts in the community composition rather than due to stoichiometric flexibility of the same community. It is well known that fungal: bacterial ratios decrease from litter and organic soils toward mineral soils (Maassen et al., 2006; Moore et al., 2010; Lee et al., 2013). For example, since both fungal: bacterial ratios and the C:N ratio of SOM decrease with soil depth, a positive correlation of microbial biomass C:N with resource C:N is expected. However, it remains unclear whether such associations between microbial biomass stoichiometry and community composition reflect a causal effect, and to what extent, or are merely coincidental due to adaptation to resource chemistry. Assessing the effect of microbial community composition on overall microbial biomass stoichiometry will therefore require more direct approaches, such as *in situ* measurements of single-cell C:N:P ratios in soils by techniques such as X-ray microanalysis or NanoSIMS coupled with phylogenetic classification at broad scale (bacteria, fungi) or fine scale (fluorescence *in situ* hybridization; Hall et al., 2011).

(2) *Microbes adjust their EE production, to maximize the mobilization of substrates rich in the limiting element*. Microbial regulation of EE production is complex, including constitutive secretion of low levels of EE (most likely to detect suitable substrates), induction of EE synthesis in response to increasing availability of complex substrates, and feedback inhibition of EE activity by their products (Wallenstein and Weintraub, 2008; Burns et al., 2013). Many EEs in soils become stabilized through association with clay minerals, humic acids and particulate organic matter, often leading to lower activities and greater residence times in soils, but such immobilized enzymes may also serve as a first sensor communicating changes in substrate availability to microbes (Burns et al., 2013). Another factor that complicates the EE response of microbes lies in the fact that enzyme production itself requires an investment of N and C, which can further increase elemental limitation (Schimel and Weintraub, 2003). This may be especially true for N, as the C:N ratio of proteins is usually much lower than microbial biomass C:N. Thus, under strong N limitation, excretion of EE to mobilize N-containing substrates may not be an adequate strategy for microbes to regulate their N homeostasis, unless the benefit of enzyme production (i.e., N released from litter or SOM through EE) outweighs the costs involved (Schimel and Weintraub, 2003). Given that EE production involves no direct P investment we do not expect similar patterns for P acquiring enzymes.

On a global scale, the stoichiometry of EE was shown to be strongly constrained, with a mean C:N:P ratio near 1:1:1 using log-transformed potential activities of hydrolytic C, N, and P acquiring enzymes (Sinsabaugh et al., 2008, 2009). However, significant variations in the stoichiometry of soil EE were found at the ecosystem scale (Sinsabaugh et al., 2008). In terms of microbial C:N:P homeostasis, we would indeed expect that EE activities are not stoichiometrically invariant to resource elemental ratios, because according to resource allocation theory microbes invest abundant elements into EE production mining for scarce elements (Allison et al., 2011). There is evidence that increasing availability of N stimulates C mobilizing enzymes (Allison and Vitousek, 2005; Geisseler and Horwath, 2009) and causes enhanced mobilization of P by enhancing phosphatase activity, i.e., an effective investment of N to mine for P in order to keep elemental balance (Olander and Vitousek, 2000; Marklein and Houlton, 2012; Mooshammer et al., 2012). In contrast, P availability did not affect N acquiring enzymes (Olander and Vitousek, 2000). In some cases, the input of labile C, which can induce N limitation in microbes, did enhance the activity of N acquiring enzymes although in other cases it did not (Allison and Vitousek, 2005; Geisseler and Horwath, 2009; Hernandez and Hobbie, 2010), which possibly depends on the overall N limitation of the microbial community.

Input of labile C was also shown to make N accessible for microbes by increased production of oxidative enzymes, resulting in enhanced SOM decomposition (“rhizosphere priming effect” or “nitrogen mining”) (Craine et al., 2007; Blagodatskaya and Kuzyakov, 2008; Sinsabaugh, 2010). In contrast to this release of N through oxidation of SOM, much of the organic P in soils is present in monoesters and diesters, which is released by hydrolysis through the activity of phosphatases (McGill and Cole, 1981; Vitousek and Howarth, 1991). Therefore, rhizosphere priming may not occur in systems that are P limited, as rhizodeposition may be utilized to mobilize P from organic and inorganic sources (through dissolution/desorption and hydrolysis, respectively), rather than for decomposition of SOM (Dijkstra et al., 2013). Overall, the available data indicate that the regulation of hydrolytic and oxidative EEs can bring nutrient and C supply closer to microbial element demand, although the general validity and relative role of this mechanism must yet be demonstrated.

If microbes indeed reduce the elemental imbalance between bulk substrate (soil or litter) and their biomass composition through the release of EE, we would expect that the C:N:P ratio of dissolved organic matter (DOM) is closer to the stoichiometry of microbial biomass than the bulk resource. However, C:N ratios of DOM (DOC:DON) in soil water and leachates are often higher than C:N ratios of SOM and are only weakly, or not significantly, related across sites (Neff et al., 2000; Wu et al., 2010; Haney et al., 2012). Soil microbial activity was nonetheless more strongly related to water-soluble organic C:N than to soil C:N in arable fields (Haney et al., 2012), which highlights that water-soluble C:N represents a more sensitive measure of the soil substrate driving microbial activity. Moreover, the bioavailability of DOM, or extractable organic matter, can be reduced (i.e., the interaction between microorganisms and DOM is restricted) by physical (e.g., inaccessibility of DOM) and chemical (e.g., DOM sorption to solid surfaces) restrictions (Marschner and Kalbitz, 2003), thus causing great differences in DOM availability between soils and soil microhabitats. For example, DOM bioavailability was found to range between 10 and 20% in organic soils (Kiikkila et al., 2005), and reached 80 to 90% in agricultural (mineral) soils (Tian et al., 2010b). Bulk measurements of C:N:P in DOM therefore also do not represent the immediate source of elements for microbes.

The composition of DOM is not only a result of the input, but is also affected by uptake through the soil microbial community, which is a virtually unexplored field given the methodological difficulties to assess the immediate substrates for microbial uptake. An inverse approach that may hold promise to dissect the elemental composition of the resource used *in situ* by soil microbes is to infer microbial C:N:P uptake from measured C:N:P ratios of mineralization fluxes, microbial biomass and bulk soil. However, this approach relies strongly on knowledge about microbial element use efficiencies (Murphy et al., 2003; Herrmann and Witter, 2008), and these are hardly ever measured for N and P (see below). An alternative approach is to determine the pools, and gross production and consumption rates of major low-molecular weight compounds that serve as the immediate substrates for heterotrophic soil microbes, including sugars, organic acids, amino acids, amino sugars, and organic phosphates, and thereby estimate the C:N:P ratios of substrates taken up by soil microbial communities.

(3) *Microbes excrete elements that are present in excess in their resource compared to their biomass composition by adjusting their element use efficiencies*. If microbes cannot change the elemental composition of their immediate substrates or adjust their biomass composition accordingly, they could take up whatever substrate is available and release elements in excess of their requirement, while keeping those in short supply for growth. Microbes are able to achieve this balance by regulating their element use efficiencies, such as the carbon use efficiency (CUE, sometimes also called growth yield or gross growth efficiency). Microbial CUE is defined as the ratio of C invested in growth (new biomass production) over total C taken up (Del Giorgio and Cole, 1998; Manzoni et al., 2012).

The considerable differences in C:N:P stoichiometry between plant detritus (litter) and SOM result in distinct elemental limitations for the respective microbial communities (**Table 1**, **Figure 1**). Progressively lower C:N and C:P ratios from litter to topsoil, and further to subsoil, correspond also to a decreasing C availability in relation to N and P (increasing C limitation). In order to cope with such differences in resource elemental composition, microbes are expected to adjust their element use efficiencies accordingly. For example, assuming strict homeostasis and negligible adjustment of EE, when microbes with a biomass C:N of 7 (global average, **Table 1**) decompose plant litter with a relatively high C:N ratio of 70, they must release 63 units of C as CO_2_ per unit of N invested in growth, yielding a low CUE of about 0.1. By contrast, if SOM with a C:N close to that of the microbial biomass would be decomposed (e.g., SOM C:N of 12 and biomass C:N of 7.2), CUE would converge toward the theoretical maximum of about 0.6 (Sinsabaugh et al., 2013). CUE cannot reach the maximum of 1, given that a significant amount of C taken up is required to produce energy for growth, maintenance and enzyme production (Schimel and Weintraub, 2003; Manzoni et al., 2012; Sinsabaugh et al., 2013). In this situation, when C becomes limiting and CUE cannot be further increased, microbes need to lower their nitrogen use efficiency (NUE), i.e., excrete N in excess (Manzoni and Porporato, 2009). This transition from net nutrient immobilization to net nutrient mineralization (critical C:N ratio) corresponds to the threshold elemental ratio (TER), which defines the transition of an ecological system from being controlled by a limiting nutrient (N or P) to being controlled by energy (C; e.g., Urabe and Watanabe, 1992; Anderson and Hessen, 1995; Frost et al., 2006). Thus, a certain nutrient becomes limiting for growth, when resource C:nutrient ratios are greater than TER.

Whereas the stoichiometric regulation of CUE has been recently reviewed in detail (Manzoni et al., 2012; Sinsabaugh et al., 2013), very little is known about regulation of microbial NUE, and even less about microbial phosphorus use efficiency (PUE) in soils. From theoretical considerations, we can postulate that, in contrast to CUE, NUE can approach the theoretical maximum of 1 if all organic N taken up is used for growth. In addition, NUE can be regulated independently from CUE, allowing microbial communities to adjust to resources with low C:N, which leads to C limitation and consequently N excess. It remains to be seen, however, if microbial NUE is also regulated at high substrate C:N ratios where microbial N limitation is expected. In such a case, microorganisms could respond to stoichiometrically unbalanced substrates by concurrent fine-tuning of CUE, NUE, and PUE, depending on the limiting element. In conclusion, the regulation of element use efficiencies is likely an important microbial strategy to cope with variations in resource stoichiometry.

Adaptations in microbial element use efficiencies have also a potentially great impact on the elemental ratios of major biogeochemical fluxes (e.g., the ratio of heterotrophic respiration to microbial N mineralization), with broad implications for soil C sequestration and N losses from terrestrial ecosystems. This means that microbial homeostasis achieved through adaptations in microbial element use efficiencies is expected to cause a strong positive relationship between resource C:N:P and mineralization flux C:N:P (CNR theory; Sterner and Elser, 2002), as the limiting elements are retained and incorporated into microbial biomass, and those in excess are excreted. In contrast, both non-homeostatic regulation of microbial biomass stoichiometry and compensatory EE regulation are expected to cause reductions in microbial element imbalances. Thus, if such a mechanism dominates, the ratios of element mineralization fluxes should not, or only slightly, increase with resource C:N:P, except when those mechanisms cannot compensate for the resource imbalance, especially with non-homeostatic regulation of microbial biomass stoichiometry at wide resource C:N:P ratios. However, the relationship between the stoichiometry of resource and mineralization fluxes as predicted by CNR theory has not yet been explicitly tested for terrestrial microbial communities.

There are few reports showing that C:N:P ratios of mineralization fluxes are strongly positively related to resource C:N:P. For instance, in decomposing litter, resource C:N and C:P were strongly negatively correlated with the respective gross N mineralization and gross P mineralization fluxes (but less so with respiration), suggestive of increasing microbial NUE and PUE at high resource C:N and C:P ratios while microbial communities were homeostatic with respect to these element ratios (Mooshammer et al., 2012). Moreover, in forest soils, Achat et al. (2010) demonstrated a similar relationship, with relatively constant microbial biomass C:P, while C:P mineralization fluxes were highly variable and strongly positively related to resource C:P [data recalculated from Achat et al. (2010)]. Both studies therefore point to the importance of regulation of microbial NUE and PUE as an adaptation to stoichiometric imbalances. Concurrent measurements of the C:N:P ratios of resources, microbial biomass, EE activities and mineralization fluxes would therefore allow deeper insights into the mechanisms used by soil microorganisms to adapt to elemental imbalances in their resources, and to constrain the most important mechanisms and their environmental controls. Addressing these questions will require advances in the measurement of CUE, NUE, and PUE in soil microbial communities.

*(4) Nitrogen-fixing prokaryotes and saprotrophic fungi increase the N and P availability by inputs from external sources*. There are two major pathways for input of N or P to the decomposing material from external sources, (i) N fixation by prokaryotes and (ii) fungal transfer of N or P from nutrient-rich patches. Prokaryotic N fixers (diazotrophs) convert atmospheric N_2_ to ammonia, a highly energy demanding process that is under strict physiological control (Raymond et al., 2004). Amongst other controls, biological N fixation is stimulated by elevated concentrations of labile organic C and P and feedback inhibited by high concentrations of ammonium and amino acids (Reed et al., 2011). Accordingly N fixation by free-living microbes is distributed heterogeneously, with hotspots of N fixation in habitats with high C and low N availability, i.e., in woody debris, leaf litter and the forest floor, and lowest rates in mineral soils (Hope and Li, 1997; Wei and Kimmins, 1998; Reed et al., 2007; Cusack et al., 2009). Moreover, release of C-rich exudates by roots also causes increased diazotroph abundances in the plant rhizosphere compared to bulk soils (Hamelin et al., 2002; Bürgmann et al., 2005). Through the release of ammonium and amino acids and upon death and lysis of diazotrophs, fixed N becomes available to other decomposers. However, though globally important amounts of N are fixed by free-living diazotrophs (Wang and Houlton, 2009), their importance to lower the C:N imbalance between resources and decomposer communities in litter has yet to be demonstrated. Diazotrophs represent only a small fraction of the decomposer community (Reed et al., 2010; Jung et al., 2012; Ducey et al., 2013) and N fixation rates are therefore orders of magnitude lower than microbial respiration rates in woody debris, leaf litter, forest floor, and mineral soils (Hope and Li, 1997; Hicks et al., 2003). Therefore the community level impact of N subsidy by diazotrophs to meet substrate C:N imbalances can be expected to be small.

Though for P there is no analogous process to N fixation, fungal mycelia may relocate P in addition to N from other sources to supplement bacterial decomposer communities at sites where these elements are scarce. Hyphae of fungal saprophytes have been shown to often extend well beyond the resource that they decompose (Strickland and Rousk, 2010) and have been demonstrated to mediate nutrient import from nutrient-rich patches into nutrient-poor habitats, e.g., from soil into decomposing litter (Osono et al., 2003; Chigineva et al., 2011) or from nutrient-rich to nutrient-poor litter (Schimel and Hättenschwiler, 2007). In addition, they may mediate reciprocal transfer of C and N between soil and litter, relocating C from litter to soil and N from soil to litter (Frey et al., 2003). Fungi thereby can significantly contribute to close the stoichiometric imbalance between resources and decomposer communities.

## CONCLUSION

In this review, we demonstrate that terrestrial microbial communities have to cope with a large spatio-temporal variability in resource stoichiometry, which can result in strong imbalances between resource composition and elemental demands by soil microbes. We highlight four major mechanisms that allow microbial communities to adapt to these environmental constraints (**Figure 1**): (i) plasticity of microbial biomass C:N:P, (ii) compensatory regulation of EE production as C and nutrient acquisition strategy, (iii) adjustments in microbial element use efficiencies and (iv) input of external nutrients by diazotrophic bacteria or saprotrophic fungi. Although the four described mechanisms clearly operate in parallel, their contributions on spatial, temporal and ecosystem scales remain underexplored. The wealth of measurements of microbial biomass C:N:P ratios sets limits to the non-homeostatic behavior of soil microbial communities. Growth of terrestrial decomposer communities on many types of resources with either very wide C:nutrient ratios (e.g., wood) or very low C:nutrient ratios (e.g., deep SOM) cannot be achieved solely through non-homeostatic behavior. Therefore, adaptation of microbial element use efficiencies and compensatory regulation of EE production are expected to contribute significantly to the adaptation of microorganisms to chemically diverse environments, together with external inputs of nutrients mediated by subgroups of the microbial community.

## Conflict of Interest Statement

The authors declare that the research was conducted in the absence of any commercial or financial relationships that could be construed as a potential conflict of interest.

## References

[B1] Achat D. L. Bakker M. R. Zeller B. Pellerin S. Bienaime S. Morel C. (2010). Long-term organic phosphorus mineralization in Spodosols under forests and its relation to carbon and nitrogen mineralization. *Soil Biol. Biochem.* 42 1479–1490 10.1016/j.soilbio.2010.05.020

[B2] Achbergerova L. Nahalka J. (2011). Polyphosphate – an ancient energy source and active metabolic regulator. *Microb. Cell Fact.* 10 63 10.1186/1475-2859-10-63 PMC3163519 21816086

[B3] Allison S. D. Vitousek P. M. (2005). Responses of extracellular enzymes to simple and complex nutrient inputs. *Soil Biol. Biochem.* 37 937–944 10.1016/j.soilbio.2004.09.014

[B4] Allison S. D. Weintraub M. N. Gartner T. B. Waldrop M. P. (2011). “Evolutionary-economic principles as regulators of soil enzyme production and ecosystem function,” in *Soil Enzymology* eds Shukla G. Varma A. (Berlin Heidelberg: Springer-Verlag) 229–243

[B5] Anderson T. R. Hessen D. O. (1995). Carbon or nitrogen limitation in marine copepods? *J. Plankton Res.* 17 317–331 10.1093/plankt/17.2.317

[B6] Banham A. H. Whatley F. R. (1991). Lack of nitrogen storage by *Paracoccus denitrificans*. *Proc. R. Soc. B Biol. Sci.* 245 211–214 10.1098/rspb.1991.0111

[B7] Bardgett R. D. Bowman W. D. Kaufmann R. Schmidt S. K. (2005). A temporal approach to linking aboveground and belowground ecology. *Trends Ecol. Evol.* 20 634–641 10.1016/j.tree.2005.08.005 16701447

[B8] Blagodatskaya E. Kuzyakov Y. (2008). Mechanisms of real and apparent priming effects and their dependence on soil microbial biomass and community structure: critical review. *Biol. Fertil. Soils* 45 115–131 10.1007/s00374-008-0334-y

[B9] Bürgmann H. Meier S. Bunge M. Widmer F. Zeyer J. (2005). Effects of model root exudates on structure and activity of a soil diazotroph community. *Environ. Microbiol.* 7 1711–1724 10.1111/j.1462-2920.2005.00818.x 16232286

[B10] Burns R. G. Deforest J. L. Marxsen J. Sinsabaugh R. L. Stromberger M. E. Wallenstein M. D. (2013). Soil enzymes in a changing environment: current knowledge and future directions. *Soil Biol. Biochem.* 58 216–234 10.1016/j.soilbio.2012.11.009

[B11] Chigineva N. I. Aleksandrova A. V. Marhan S. Kandeler E. Tiunov A. V. (2011). The importance of mycelial connection at the soil-litter interface for nutrient translocation, enzyme activity and litter decomposition. *Appl. Soil Ecol.* 51 35–41 10.1016/j.apsoil.2011.08.009

[B12] Cleveland C. C. Liptzin D. (2007). C:N:P stoichiometry in soil: is there a “Redfield ratio” for the microbial biomass? *Biogeochemistry* 85 235–252 10.1007/s10533-007-9132-0

[B13] Craine J. M. Morrow C. Fierer N. (2007). Microbial nitrogen limitation increases decomposition. *Ecology* 88 2105–2113 10.1890/06-1847.1 17824441

[B14] Cusack D. F. Silver W. Mcdowell W. H. (2009). Biological nitrogen fixation in two tropical forests: ecosystem-level patterns and effects of nitrogen fertilization. *Ecosystems* 12 1299–1315 10.1007/s10021-009-9290-0

[B15] Del Giorgio P. A. Cole J. J. (1998). Bacterial growth efficiency in natural aquatic systems. *Annu. Rev. Ecol. Syst.* 29 503–541 10.1146/annurev.ecolsys.29.1.503

[B16] Dijkstra F. A. Carrillo Y. Pendall E. Morgan J. A. (2013). Rhizosphere priming: a nutrient perspective. *Front. Microbiol.* 4:216 10.3389/fmicb.2013.00216 PMC3725428 23908649

[B17] Drake J. E. Darby B. A. Giasson M. A. Kramer M. A. Phillips R. P. Finzi A. C. (2013). Stoichiometry constrains microbial response to root exudation-insights from a model and a field experiment in a temperate forest. *Biogeosciences* 10 821–838 10.5194/bg-10-821-2013

[B18] Drake J. E. Gallet-Budynek A. Hofmockel K. S. Bernhardt E. S. Billings S. A. Jackson R. B. (2011). Increases in the flux of carbon belowground stimulate nitrogen uptake and sustain the long-term enhancement of forest productivity under elevated CO_2_. *Ecol. Lett.* 14 349–357 10.1111/j.1461-0248.2011.01593.x 21303437

[B19] Ducey T. F. Ippolito J. A. Cantrell K. B. Novak J. M. Lentz R. D. (2013). Addition of activated switchgrass biochar to an aridic subsoil increases microbial nitrogen cycling gene abundances. *Appl. Soil Ecol.* 65 65–72 10.1016/j.apsoil.2013.01.006

[B20] Egli T. (1995). The ecological and physiological significance of the growth of heterotrophic microorganisms with mixtures of substrates. *Adv. Microb. Ecol.* 14 305

[B21] Elser J. J. Acharya K. Kyle M. Cotner J. Makino W. Markow T. (2003). Growth rate-stoichiometry couplings in diverse biota. *Ecol. Lett.* 6 936–943 10.1046/j.1461-0248.2003.00518.x

[B22] Elser J. J. Urabe J. (1999). The stoichiometry of consumer-driven nutrient recycling: theory, observations, and consequences. *Ecology* 80 735–751 10.1890/0012-9658(1999)080[0735:TSOCDN]2.0.CO;2

[B23] Fanin N. Fromin N. Buatois B Hättenschwiler S. (2013). An experimental test of the hypothesis of non-homeostatic consumer stoichiometry in a plant litter-microbe system. *Ecol. Lett.* 16 764–772 10.1111/ele.12108 23521784

[B24] Fierer N. Bradford M. A. Jackson R. B. (2007). Toward an ecological classification of soil bacteria. *Ecology* 88 1354–1364 10.1890/05-1839 17601128

[B25] Frey S. D. Six J. Elliott E. T. (2003). Reciprocal transfer of carbon and nitrogen by decomposer fungi at the soil-litter interface. *Soil Biol. Biochem.* 35 1001–1004 10.1016/S0038-0717(03)00155-X

[B26] Frost P. C. Benstead J. P. Cross W. F. Hillebrand H. Larson J. H. Xenopoulos M. A. (2006). Threshold elemental ratios of carbon and phosphorus in aquatic consumers. *Ecol. Lett.* 9 774–779 10.1111/j.1461-0248.2006.00919.x 16796566

[B27] Geisseler D. Horwath W. R. (2009). Relationship between carbon and nitrogen availability and extracellular enzyme activities in soil. *Pedobiologia* 53 87–98 10.1016/j.pedobi.2009.06.002

[B28] Hall E. K. Maixner F. Franklin O. Daims H. Richter A. Battin T. (2011). Linking microbial and ecosystem ecology using ecological stoichiometry: a synthesis of conceptual and empirical approaches. *Ecosystems* 14 261–273 10.1007/s10021-010-9408-4

[B29] Hamelin J. Fromin N. Tarnawski S. Teyssier-Cuvelle S. Aragno M. (2002). nifH gene diversity in the bacterial community associated with the rhizosphere of *Molinia coerulea*, an oligonitrophilic perennial grass. *Environ. Microbiol.* 4 477–481 10.1046/j.1462-2920.2002.00319.x 12153588

[B30] Haney R. L. Franzluebbers A. J. Jin V. L. Johnson M.-V. Haney E. B. White M. J. (2012). Soil organic C:N vs. water-extractable organic C:N. *Open J. Sci.* 2 269–274 10.4236/ojss.2012.23032

[B31] Harmon M. E. Franklin J. F. Swanson F. J. Sollins P. Gregory S. V. Lattin J. D. (1986). Ecology of coarse woody debris in temperate ecosystems. *Adv. Ecol. Res.* 15 133–302 10.1016/S0065-2504(08)60121-X

[B32] Hartman W. H. Richardson C. J. (2013). Differential nutrient limitation of soil microbial biomass and metabolic quotients (*q*CO_2_): is there a biological stoichiometry of soil microbes? *PLoS ONE* 8:e57127 10.1371/journal.pone.0057127 PMC3602520 23526933

[B33] Hernandez D. L. Hobbie S. E. (2010). The effects of substrate composition, quantity, and diversity on microbial activity. *Plant Soil* 335 397–411 10.1007/s11104-010-0428-9

[B34] Herrmann A. M. Witter E. (2008). Predictors of gross N mineralization and immobilization during decomposition of stabilized organic matter in agricultural soil. *Eur. J. Soil Sci.* 59 653–664 10.1111/j.1365-2389.2008.01023.x

[B35] Hicks W. T. Harmon M. E. Myrold D. D. (2003). Substrate controls on nitrogen fixation and respiration in woody debris from the Pacific Northwest, USA. *For. Ecol. Manage.* 176 25–35 10.1016/S0378-1127(02)00229-3

[B36] Hope S. M. Li C. Y. (1997). Respiration, nitrogen fixation, and mineralizable nitrogen spatial and temporal patterns within two Oregon Douglas-fir stands. *Can. J. For. Res.* 27 501–509

[B37] Jung J. Yeom J. Han J. Kim J. Park W. (2012). Seasonal changes in nitrogen-cycle gene abundances and in bacterial communities in acidic forest soils. *J. Microbiol.* 50 365–373 10.1007/s12275-012-1465-2 22752898

[B38] Kiikkila O. Kitunen V. Smolander A. (2005). Degradability of dissolved soil organic carbon and nitrogen in relation to tree species. *FEMS Microbiol. Ecol.* 53 33–40 10.1016/j.femsec.2004.08.011 16329927

[B39] Kornberg A. (1995). Inorganic polyphosphate – toward making a forgotten polymer unforgettable. *J. Bacteriol.* 177 491–496 7836277 10.1128/jb.177.3.491-496.1995 PMC176618

[B40] Lee S. H. Jang I. Chae N. Choi T. Kang H. (2013). Organic layer serves as a hotspot of microbial activity and abundance in arctic tundra soils. *Microb. Ecol.* 65 405–414 10.1007/s00248-012-0125-8 22983497

[B41] Lee S. Y. (1996). Bacterial polyhydroxyalkanoates. *Biotechnol. Bioeng.* 49 1–14 10.1002/(SICI)1097-0290(19960105)49:1<1::AID-BIT1>3.3.CO;2-1 18623547

[B42] Li Y. Wu J. Liu S. Shen J. Huang D. Su Y. (2012). Is the C:N:P stoichiometry in soil and soil microbial biomass related to the landscape and land use in southern subtropical China? *Global Biogeochemical Cycles* 26 10.1029/2012GB004399

[B43] Maassen S. Fritze H. Wirth S. (2006). Response of soil microbial biomass, activities, and community structure at a pine stand in northeastern Germany 5 years after thinning. *Can. J. For. Res. Rev.* 36 1427–1434 10.1139/x06-039

[B44] Makino W. Cotner J. B. Sterner R. W. Elser J. J. (2003). Are bacteria more like plants or animals? Growth rate and resource dependence of bacterial C : N : P stoichiometry. *Funct. Ecol.* 17 121–130 10.1046/j.1365-2435.2003.00712.x

[B45] Manzoni S. Porporato A. (2009). Soil carbon and nitrogen mineralization: theory and models across scales. *Soil Biol. Biochem.* 41 1355–1379 10.1016/j.soilbio.2009.02.031

[B46] Manzoni S. Taylor P. Richter A. Porporato A Ågren G. I. (2012). Environmental and stoichiometric controls on microbial carbon-use efficiency in soils. *New Phytol.* 196 79–91 10.1111/j.1469-8137.2012.04225.x 22924405

[B47] Manzoni S. Trofymow J. A. Jackson R. B. Porporato A. (2010). Stoichiometric controls on carbon, nitrogen, and phosphorus dynamics in decomposing litter. *Ecol. Monogr.* 80 89–106 10.1890/09-0179.1

[B48] Marklein A. R. Houlton B. Z. (2012). Nitrogen inputs accelerate phosphorus cycling rates across a wide variety of terrestrial ecosystems. *New Phytol.* 193 696–704 10.1111/j.1469-8137.2011.03967.x 22122515

[B49] Marschner B. Kalbitz K. (2003). Controls of bioavailability and biodegradability of dissolved organic matter in soils. *Geoderma* 113 211–235 10.1016/S0016-7061(02)00362-2

[B50] Marschner H. (1995). *Mineral Nutrition of Higher Plants*. London: Academic Press

[B51] Martinelli L. A. Almeida S. Brown I. F. Moreira M. Z. Victoria R. L. Filoso S. (2000). Variation in nutrient distribution and potential nutrient losses by selective logging in a humid tropical forest of Rondonia, Brazil. *Biotropica* 32 597–613 10.1646/0006-3606(2000)032[0597:VINDAP]2.0.CO;2

[B52] McGill W. B. Cole C. V. (1981). Comparative aspects of cycling of organic C, N, S and P through soil organic matter. *Geoderma* 26 267–286 10.1016/0016-7061(81)90024-0

[B53] McGroddy M. E. Daufresne T. Hedin L. O. (2004). Scaling of C:N:P stoichiometry in forests worldwide: implications of terrestrial redfield-type ratios. *Ecology* 85 2390–2401 10.1890/03-0351

[B54] Miltner A. Kindler R. Knicker H. Richnow H.-H. Kaestner M. (2009). Fate of microbial biomass-derived amino acids in soil and their contribution to soil organic matter. *Org. Geochem.* 40 978–985 10.1016/j.orggeochem.2009.06.008

[B55] Moore J. Macalady J. L. Schulz M. S. White A. F. Brantley S. L. (2010). Shifting microbial community structure across a marine terrace grassland chronosequence, Santa Cruz, California. *Soil Biol. Biochem.* 42 21–31 10.1016/j.soilbio.2009.09.015

[B56] Moorhead D. L. Lashermes G. Sinsabaugh R. L. (2012). A theoretical model of C- and N-acquiring exoenzyme activities, which balances microbial demands during decomposition. *Soil Biol. Biochem.* 53 133–141 10.1016/j.soilbio.2012.05.011

[B57] Mooshammer M. Wanek W. Schnecker J. Wild B. Leitner S. Hofhansl F. (2012). Stoichiometric controls of nitrogen and phosphorus cycling in decomposing beech leaf litter. *Ecology* 93 770–782 10.1890/11-0721.1 22690628

[B58] Murphy D. V. Recous S. Stockdale E. A. Fillery I. R. P. Jensen L. S. Hatch D. J. (2003). Gross nitrogen fluxes in soil: theory, measurement and application of 15N pool dilution techniques. *Adv. Agron.* 79 69–118 10.1016/S0065-2113(02)79002-0

[B59] Neff J. C. Hobbie S. E. Vitousek P. M. (2000). Nutrient and mineralogical control on dissolved organic C, N and P fluxes and stoichiometry in Hawaiian soils. *Biogeochemistry* 51 283–302 10.1023/A:1006414517212

[B60] Olander L. P. Vitousek P. M. (2000). Regulation of soil phosphatase and chitinase activity by N and P availability. *Biogeochemistry* 49 175–190 10.1023/A:1006316117817

[B61] Osono T. Ono Y. Takeda H. (2003). Fungal ingrowth on forest floor and decomposing needle litter of *Chamaecyparis obtusa* in relation to resource availability and moisture condition. *Soil Biol. Biochem.* 35 1423–1431 10.1016/S0038-0717(03)00236-0

[B62] Phillips R. P. Finzi A. C. Bernhardt E. S. (2011). Enhanced root exudation induces microbial feedbacks to N cycling in a pine forest under long-term CO2 fumigation. *Ecol. Lett.* 14 187–194 10.1111/j.1461-0248.2010.01570.x 21176050

[B63] Raymond J. Siefert J. L. Staples C. R. Blankenship R. E. (2004). The natural history of nitrogen fixation. *Mol. Biol. Evol.* 21 541–554 10.1093/molbev/msh047 14694078

[B64] Reed S. C. Cleveland C. C. Townsend A. R. (2007). Controls over leaf litter and soil nitrogen fixation in two lowland tropical rain forests. *Biotropica* 39 585–592 10.1111/j.1744-7429.2007.00310.x

[B65] Reed S. C. Cleveland C. C. Townsend A. R. (2011). Functional ecology of free-living nitrogen fixation: a contemporary perspective. *Annu. Rev. Ecol. Evol. Syst.* 42 489–512 10.1146/annurev-ecolsys-102710-145034

[B66] Reed S. C. Townsend A. R. Cleveland C. C. Nemergut D. R. (2010). Microbial community shifts influence patterns in tropical forest nitrogen fixation. *Oecologia* 164 521–531 10.1007/s00442-010-1649-6 20454976

[B67] Schimel J. P. Bennett J. (2004). Nitrogen mineralization: challenges of a changing paradigm. *Ecology* 85 591–602 10.1890/03-8002

[B68] Schimel J. P Hättenschwiler S. (2007). Nitrogen transfer between decomposing leaves of different N status. *Soil Biol. Biochem.* 39 1428–1436 10.1016/j.soilbio.2006.12.037

[B69] Schimel J. P. Weintraub M. N. (2003). The implications of exoenzyme activity on microbial carbon and nitrogen limitation in soil: a theoretical model. *Soil Biol. Biochem.* 35 549–563 10.1016/S0038-0717(03)00015-4

[B70] Scott J. T. Cotner J. B. Lapara T. M. (2012). Variable stoichiometry and homeostatic regulation of bacterial biomass elemental composition. *Front. Microbiol.* 3:42. 10.3389/fmicb.2012.00042 PMC3283892 22371708

[B71] Simpson A. J. Simpson M. J. Smith E. Kelleher B. P. (2007). Microbially derived inputs to soil organic matter: are current estimates too low? *Environ. Sci. Technol.* 41 8070–8076 10.1021/es071217x 18186339

[B72] Sinsabaugh R. L. (2010). Phenol oxidase, peroxidase and organic matter dynamics of soil. *Soil Biol. Biochem.* 42 391–404 10.1016/j.soilbio.2009.10.014

[B73] Sinsabaugh R. L. Hill B. H Shah J. J. F. (2009). Ecoenzymatic stoichiometry of microbial organic nutrient acquisition in soil and sediment. *Nature* 462 795–798 10.1038/nature08632 20010687

[B74] Sinsabaugh R. L. Lauber C. L. Weintraub M. N. Ahmed B. Allison S. D. Crenshaw C. (2008). Stoichiometry of soil enzyme activity at global scale. *Ecol. Lett.* 11 1252–1264 10.1111/j.1461-0248.2008.01245.x 18823393

[B75] Sinsabaugh R. L. Manzoni S. Moorhead D. L. Richter A. (2013). Carbon use efficiency of microbial communities: stoichiometry, methodology and modelling. *Ecol. Lett.* 16 930–939 10.1111/ele.12113 23627730

[B76] Sistla S. A. Schimel J. P. (2012). Stoichiometric flexibility as a regulator of carbon and nutrient cycling in terrestrial ecosystems under change. *New Phytol.* 196 68–78 10.1111/j.1469-8137.2012.04234.x 22924404

[B77] Sterner R. W. (1990). The ratio of nitrogen to phosphorus resupplied by herbivores: zooplankton and the algal competitive arena. *Am. Nat.* 136 209–229 10.1086/285092

[B78] Sterner R. W. Elser J. J. (2002). *Ecological Stoichiometry: The Biology of Elements from Molecules to the Biosphere*. Princeton: Princeton University Press

[B79] Strickland M. S. Rousk J. (2010). Considering fungal:bacterial dominance in soils – Methods, controls, and ecosystem implications. *Soil Biol. Biochem.* 42 1385–1395 10.1016/j.soilbio.2010.05.007

[B80] Tian H. Q. Chen G. S. Zhang C. Melillo J. M Hall C. A. S. (2010a). Pattern and variation of C:N:P ratios in China’s soils: a synthesis of observational data. *Biogeochemistry* 98 139–151 10.1007/s10533-009-9382-0

[B81] Tian L. Dell E. Shi W. (2010b). Chemical composition of dissolved organic matter in agroecosystems: correlations with soil enzyme activity and carbon and nitrogen mineralization. *Appl. Soil Ecol.* 46 426–435 10.1016/j.apsoil.2010.09.007

[B82] Urabe J. Watanabe Y. (1992). Possibility of N or P limitation for planktonic cladocerans: an experimental test. *Limnol. Oceanog.* 37 244–251 10.4319/lo.1992.37.2.0244

[B83] Vitousek P. M. Howarth R. W. (1991). Nitrogen limitation on land and in the sea: how can it occur? *Biogeochemistry* 13 87–115 10.1007/BF00002772

[B84] Wallenstein M. D. Weintraub M. N. (2008). Emerging tools for measuring and modeling the in situ activity of soil extracellular enzymes. *Soil Biol. Biochem.* 40 2098–2106 10.1016/j.soilbio.2008.01.024

[B85] Wang Y. P. Houlton B. Z. (2009). Nitrogen constraints on terrestrial carbon uptake: implications for the global carbon-climate feedback. *Geophys. Res. Lett.* 36 24 10.1029/2009GL041009

[B86] Weedon J. T. Cornwell W. K. Cornelissen J. H. C. Zanne A. E. Wirth C. Coomes D. A. (2009). Global meta-analysis of wood decomposition rates: a role for trait variation among tree species? *Ecol. Lett.* 12 45–56 10.1111/j.1461-0248.2008.01259.x 19016827

[B87] Wei X. Kimmins J. P. (1998). Asymbiotic nitrogen fixation in harvested and wildfire-killed lodgepole pine forests in the central interior of British Columbia. *For. Ecol. Manag.* 109 343–353 10.1016/S0378-1127(98)00288-6

[B88] Wilkinson J. F. (1963). Carbon and energy storage in bacteria. *J. Gen. Microbiol.* 32 171–176 10.1099/00221287-32-2-171 14053264

[B89] Wilson W. A. Roach P. J. Montero M. Baroja-Fernandez E. Munoz F. J. Eydallin G. (2010). Regulation of glycogen metabolism in yeast and bacteria. *FEMS Microbiol. Rev.* 34 952–985 10.1111/j.1574-6976.2010.00220.x 20412306 PMC2927715

[B90] Wu Y. J. Clarke N. Mulder J. (2010). Dissolved organic nitrogen concentrations and ratios of dissolved organic carbon to dissolved organic nitrogen in throughfall and soil waters in Norway spruce and Scots pine forest stands throughout Norway. *Water Air Soil Pollut.* 210 171–186 10.1007/s11270-009-0239-x

[B91] Xu X. Thornton P. E. Post W. M. (2013). A global analysis of soil microbial biomass carbon, nitrogen and phosphorus in terrestrial ecosystems. *Glob. Ecol. Biogeogr.* 22 737–749 10.1111/geb.12029

[B92] Yuan Z. Y. Chen H. Y. H. Reich P. B. (2011). Global-scale latitudinal patterns of plant fine-root nitrogen and phosphorus. *Nat. Commun.* 2 344 10.1038/ncomms1346 21673665

[B93] Yuan Z. Y. Y Chen H. Y. H. (2009). Global trends in senesced-leaf nitrogen and phosphorus. *Glob. Ecol. Biogeogr.* 18 532–542 10.1111/j.1466-8238.2009.00474.x

